# Apgar score and neonatal mortality in China: an observational study from a national surveillance system

**DOI:** 10.1186/s12884-020-03533-3

**Published:** 2021-01-12

**Authors:** Yi Mu, Mingrong Li, Jun Zhu, Yanping Wang, Aiyun Xing, Zheng Liu, Yanxia Xie, Xiaodong Wang, Juan Liang

**Affiliations:** 1 National Office for Maternal and Child Health Surveillance of China, West China Second University Hospital, Sichuan University, Ren Min South Road Section 3 No. 17, Chengdu, Sichuan China; 2 Key Laboratory of Birth Defects and Related Diseases of Women and Children (Sichuan University), Ministry of Education, Ren Min South Road Section 3 No. 17, Chengdu, Sichuan China; 3 Department of Obstetrics, West China Second University Hospital, Sichuan University, Ren Min South Road Section 3 No. 17, Chengdu, Sichuan China

**Keywords:** Apgar score, Neonatal mortality, Small for gestational age, China

## Abstract

**Background:**

To examine the association between the Apgar score and neonatal mortality over gestational age in China and to explore whether this association changed when Apgar scores were combined at 1 and 5 min.

**Methods:**

Data for all singleton live births collected from 438 hospitals between 2012 and 2016 were used in this study. Poisson regression with a robust variance estimator adjusted for a complete set of confounders was used to describe the strength of the association between the Apgar score and neonatal mortality.

**Results:**

The relative risks of neonatal death-associated intermediate Apgar score at 5 min peaked at 39–40 weeks of gestation and subsequently decreased if the gestational age increased to 42 weeks or above, in contrast to the low Apgar score. Among both preterm and term new-borns with Apgar scores at 5 min, new-borns that were not small for gestational age had a lower mortality rate than those that were small for gestational age. The association between Apgar score and the neonatal mortality was even stronger when scores at 1 and 5 min were combined.

**Conclusions:**

Apgar score is not only meaningful for preterm new-borns but also useful for term new-borns, especially term new-borns that are not small for gestational age. Once the baby’s Apgar score worsens, timely intervention is needed. There is still a gap between China and high-income countries in terms of sustained treatment of new-borns with low Apgar scores.

**Supplementary Information:**

The online version contains supplementary material available at 10.1186/s12884-020-03533-3.

## Background

China’s national under-five mortality rate (U5MR) declined from 61 per 1000 live births in 1991 to 11 per 1000 live births in 2015 [1], being well ahead of the target for 2015 (20.3 per 1000 live births) set by the Millennium Development Goals. Among all under-five deaths in 2016, 73.8% were concentrated in the infant group (age < 1 year), and 47.9% were neonatal deaths [2]. Preterm birth complications (mainly premature delivery or low birth weight), intrapartum-related complications (mainly birth asphyxia) and congenital abnormalities were the three major causes of neonatal death in China [3]. Neonatal resuscitation in immediate new-born care plays a very important and effective role in improving birth asphyxia-related outcomes [4]. At present, nearly all women in China choose to give birth in hospitals; thus, new-borns can receive good care from healthcare workers to minimize the risk of early neonatal mortality due to avoidable causes, such as birth asphyxia. After the Chinese government released its two-child policy in October 2015 [5], many Chinese couples were allowed and encouraged to have a second child from January 1, 2016. There is an urgent need to evaluate whether the existing neonatal status assessment methods are good enough to assist obstetricians and neonatal paediatricians in assessing the needs of obstetricians and neonatologists because of the rapid increase in the number of births.

The Apgar score has been used to quickly evaluate the physical condition of new-borns after delivery for more than 60 years [6] and is routinely used in obstetrics for every delivery in China. Nevertheless, Apgar scores are often used in the rapid assessment of asphyxia severity in clinical practice in China, although most experts have indicated that Apgar scores should not be used alone to diagnose birth asphyxia, as pointed out in Chinese textbooks of paediatrics [7]. Studies have shown that the Apgar score at 5 min after birth is related to neonatal mortality [8]. A cohort study of the UK from 1992 to 2010 suggested that the mortality rate increased with declining Apgar score at 5 min [9]. However, there are differences in neonatal resuscitation capacity, human resources, and ethics group between China and high-income countries. Whether the association between Apgar scores and neonatal mortality was different in China was uncertain, and the effect of Apgar score in terms of the difference between scores at 1 min and 5 min on neonatal mortality was not shown. Gestational age and birthweight are important indicators in predicting the health status of new-borns, but the association between neonatal mortality and Apgar score stratified by both gestational age and birthweight has also not been reported.

In this study, we first analysed data collected in over 400 hospitals from 2012 to 2016 in China to characterize maternal characteristics and delivery information in relation to Apgar scores at 5 min after birth. Second, we evaluated whether there is a correlation between Apgar score at 5 min and neonatal mortality in China and whether the association differed after adjusting for a complete set of covariates from different gestational age groups. We stratified the analysis by small for gestational age (SGA) categories to further understand the association between neonatal mortality and Apgar score at 5 min in different gestational age groups. The Apgar score is generally calculated at one and 5 min after birth; thus, the effect of the Apgar score on neonatal mortality was examined when scores at both 1 min and 5 min were combined.

## Methods

### Data sources

Data used in the study were from China’s National Maternal Near Miss Surveillance System (NMNMSS), covering 441 sampled hospitals selected from 30 provinces in China. The sampling details have been reported elsewhere [10, 11]. Within each of the sampled districts or counties, two health facilities with more than 1000 deliveries per year were selected (or one facility if only one was available). Because some districts or counties did not have hospitals with the necessary number of births, large hospitals in urban districts were oversampled. As a result, urban populations were overrepresented in the NMNMSS, particularly in the central and western regions.

For each pregnant woman or woman who was admitted to surveillance facilities up to 42 days postpartum, sociodemographic and obstetric information was collected prospectively from admission to discharge. Foetal information was also collected, including weight at birth, Apgar score and status of life. The NMNMSS was designed to enumerate all maternal deaths and near misses (women who nearly died from a severe complication of pregnancy or delivery) in health facilities based on an individual questionnaire, meaning that the life status of the new-born could only be tracked prospectively before the mother was discharged from the hospital. Data on the new-born’s life status were collected in three ways: evaluation of maternal medical records after discharge of the mother if she gave birth in the monitoring hospital; verbal inquiry of the mother or her family if she gave birth in another place; or evaluation of the infant’s medical records if the infant was transferred to the neonatology department. The data provided to us were de-identified.

### Definitions

We obtained NMNMSS data for all new-borns delivered in 438 hospitals (3 hospitals excluded because data were not reported since 2012) between Jan 1, 2012, and Dec 31, 2016. Because a report from The New England Journal of Medicine showed that the survival rate reached 81% among those born at 26 weeks of gestation [12], our analysis was restricted to singleton births born alive with a gestational age at delivery equal to or greater than 26 weeks. The gestational age in China is generally ascertained on the basis of the last menstrual period or ultrasound when the date of the last menstrual period is not known or when the menstrual cycle is irregular. The current gestational age is recorded in the maternal health booklet at each antenatal visit of a pregnant woman. We classified Apgar scores into three groups: low (Apgar 0–3), intermediate (Apgar 4–6), and normal (Apgar 7–10). The outcome was neonatal death (from birth to the date when the mother was discharged from the hospital). We excluded babies whose mothers had remained in the hospital after delivery for more than 27 days to ensure that all death cases in the study were neonatal deaths. We excluded babies delivered from abortion, including spontaneous abortion, induced abortion and medical abortion. In China, the government recommends five or more antenatal visits in rural areas and eight or more in urban areas, so the number of antenatal care visits during pregnancy was categorized as 0, 1–4, 5–7, or ≥ 8. Preterm was defined as a live birth at less than 37 weeks of gestation but equal to or more than 26 completed weeks, term was defined as a live birth between 37 and 41 completed weeks, and post-term was defined as a live birth at 42 or more gestational weeks. Standard definitions were used for low birthweight (< 2500 g) [13], normal birthweight (2500–4000 g), and macrosomia (≥4000 g) [14]. SGA was defined as weighing less than the 10th percentile based on gestational age-specific birth weight percentiles for male and female infants in China [15, 16]. We also used a second definition for SGA according to the global INTERGROWTH-21st standards [17] and compared the results between the Chinese standards and the global standards. We classified maternal complications into mutually exclusive categories of direct obstetric complications and medical diseases. Direct obstetric complications included uterine rupture, placenta praevia, abruptio placentae, unspecified antepartum haemorrhage, pre-eclampsia, eclampsia, HELLP syndrome or any foetal malpresentation (breech, shoulder or other). Medical diseases included heart disease, embolism/thrombophlebitis, hepatic disease, severe anaemia (haemoglobin < 70 g/L), renal disease (including urinary tract infection), lung disease (including upper respiratory tract infection), HIV/AIDS, connective tissue disorders, gestational diabetes mellitus and cancer.

### Statistical analysis

The alluvial diagram was used to show the association of new-borns with low and intermediate Apgar scores at 5 min and neonatal deaths under different types of maternal complications. Since the NMNMSS oversampled large urban hospitals, we weighed the neonatal mortality rates for the sampling distribution of the population according to the 2010 census of China, as detailed elsewhere [10, 11, 18]. Relative risks (RRs) and 95% confidence intervals (CIs) were used to describe the strength of the association between the Apgar score and neonatal mortality. RRs were calculated using a Poisson regression with a robust variance estimator adjusted for a complete set of confounders [19], taking into account the clustering of live births within monitoring facilities. The “ggalluvial” package of R (version 3.6.1) was used to produce the alluvial diagrams. All other analyses were performed with Stata (version 15.1).

### Ethics approval

This study was approved by the ethics committee of the West China Second University Hospital (protocol ID, 2012008).

## Results

From Jan 1, 2012, to Dec 31, 2016, the NMNMSS recorded 6,620,684 singleton live births with at least 26 completed gestational weeks. 7633 deaths occurred before the mother was discharged from the hospital, which gave a neonatal mortality rate of 0.11% after adjusting for the sampling distribution of the population.

Table 1 shows the maternal characteristics and delivery details in relation to Apgar score at 5 min. A vast majority of neonates (6,531,945, 99.66%) had a normal Apgar score at 5 min after birth. Compared with women who gave birth to infants with a normal Apgar score at 5 min (7–10), mothers of infants with a low Apgar score (0–3) or intermediate Apgar score (4–6) at 5 min were more likely to be young (under 20 years old) or of advanced maternal age (over 35 years old), to have a lower educational level, to have delivered more than once, to have received few antenatal visits, and to have used general anaesthesia during childbirth. Compared with neonates whose Apgar scores at 5 min were 7–10, neonates with low Apgar scores or intermediate Apgar scores also had a much larger proportion of non-cephalic presentation, premature birth, and low birthweight (< 2500 g).

**Table 1:** Maternal characteristics and delivery information in relation to Apgar scores at 5 min after birth

	Low Apgar score (***n*** = 9011)	Intermediate Agar score (***n*** = 13,186)	Normal Apgar score (***n*** = 6,531,945)
**Maternal age**			
< 20	421 (4.7%)	709 (5.4%)	193,249 (3.0%)
20–24	1994 (22.1%)	3057 (23.2%)	1,448,641 (22.2%)
25–29	3236 (35.9%)	4129 (31.3%)	2,728,175 (41.8%)
30–34	1745 (19.4%)	2649 (20.1%)	1,364,292 (20.9%)
35–39	831 (9.2%)	1357 (10.3%)	473,231 (7.2%)
≥ 40	241 (2.7%)	467 (3.5%)	104,635 (1.6%)
Missing	543 (6.0%)	818 (6.2%)	219,722 (3.4%)
**Maternal education degree**			
College or above	2318 (25.7%)	2727 (20.7%)	2,140,931 (32.8%)
High school	2356 (26.1%)	3371 (25.6%)	1,741,818 (26.7%)
Middle school	3409 (37.8%)	5466 (41.5%)	2,256,766 (34.5%)
Primary school or below	761 (8.4%)	1330 (10.1%)	251,327 (3.8%)
Missing	167 (1.9%)	292 (2.2%)	141,103 (2.2%)
**Marital status**			
Married	164 (1.8%)	308 (2.3%)	95,208 (1.5%)
Single, widowed or divorced	8844 (98.1%)	12,877 (97.7%)	6,435,424 (98.5%)
Missing	3 (0.0%)	1 (0.0%)	1313 (0.0%)
**Parity**			
0	4838 (53.7%)	6932 (52.6%)	3,859,354 (59.1%)
1	3320 (36.8%)	4848 (36.8%)	2,289,761 (35.1%)
≥ 2	849 (9.4%)	1402 (10.6%)	380,674 (5.8%)
Missing	4 (0.0%)	4 (0.0%)	2156 (0.0%)
**Previous CS**			
0	7624 (84.6%)	11,295 (85.7%)	5,636,817 (86.3%)
1	1240 (13.8%)	1709 (13.0%)	842,258 (12.9%)
≥ 2	137 (1.5%)	171 (1.3%)	48,241 (0.7%)
Missing	10 (0.1%)	11 (0.1%)	4629 (0.1%)
**Antenatal care**			
0	412 (4.6%)	518 (3.9%)	97,681 (1.5%)
1–4	2268 (25.2%)	3816 (28.9%)	946,945 (14.5%)
5–7	2998 (33.3%)	4744 (36.0%)	2,158,065 (33.0%)
≥ 8	3042 (33.8%)	3458 (26.2%)	3,159,318 (48.4%)
Missing	291 (3.2%)	650 (4.9%)	169,936 (2.6%)
**Mode of delivery**			
Vaginal delivery	4913 (54.5%)	7041 (53.4%)	3,618,402 (55.4%)
Caesarean section	4098 (45.5%)	6144 (46.6%)	2,913,315 (44.6%)
Missing	0 (0.0%)	1 (0.0%)	228 (0.0%)
**Fetal presentation**			
Non-cephalic	1092 (12.1%)	1776 (13.5%)	211,988 (3.2%)
Cephalic	7913 (87.8%)	11,396 (86.4%)	6,317,695 (96.7%)
Missing	6 (0.1%)	14 (0.1%)	2262 (0.0%)
**Gestational age**			
Preterm	3540 (39.3%)	6811 (51.7%)	381,772 (5.8%)
Term	5368 (59.6%)	6178 (46.9%)	6,089,384 (93.2%)
Post-term	103 (1.1%)	197 (1.5%)	60,789 (0.9%)
**Birthweight**			
Low birthweight	3309 (36.7%)	6594 (50.0%)	282,757 (4.3%)
Normal birthweight	5092 (56.5%)	5970 (45.3%)	5,791,738 (88.7%)
Macrosomia	384 (4.3%)	465 (3.5%)	447,697 (6.9%)
Missing	226 (2.5%)	157 (1.2%)	9753 (0.1%)
**Infant sex**			
Female	4043 (44.9%)	5555 (42.1%)	3,120,972 (47.8%)
Male	4912 (54.5%)	7590 (57.6%)	3,405,994 (52.1%)
Missing	56 (0.6%)	41 (0.3%)	4979 (0.1%)
**Use of anesthesia**			
None	4544 (50.4%)	6582 (49.9%)	3,282,751 (50.3%)
General anesthesia	475 (5.3%)	845 (6.4%)	65,873 (1.0%)
Only epidural anesthesia	1659 (18.4%)	2543 (19.3%)	1,305,472 (20.0%)
Combined spinal-epidural anesthesia	1976 (21.9%)	2695 (20.4%)	1,634,865 (25.0%)
Missing	357 (4.0%)	521 (4.0%)	242,984 (3.7%)
**Hospital level**			
Level 1	361 (4.0%)	468 (3.5%)	331,618 (5.1%)
Level 2	431 (4.8%)	697 (5.3%)	450,919 (6.9%)
Level 3	4026 (44.7%)	5462 (41.4%)	3,142,398 (48.1%)
Unknown*	4193 (46.5%)	6559 (49.7%)	2,607,010 (39.9%)
**Maternal complications**			
Direct obstetric complications	2343 (26.0%)	4258 (32.3%)	421,734 (6.5%)
Medical diseases	538 (6.0%)	797 (6.0%)	407,840 (6.2%)
None above	6130 (68.0%)	8131 (61.7%)	5,702,371 (87.3%)

A total of 66,542 (1.01%) cases had no information on the Apgar score at 5 min

*These hospitals did not get level certification

**Fig. 1:**
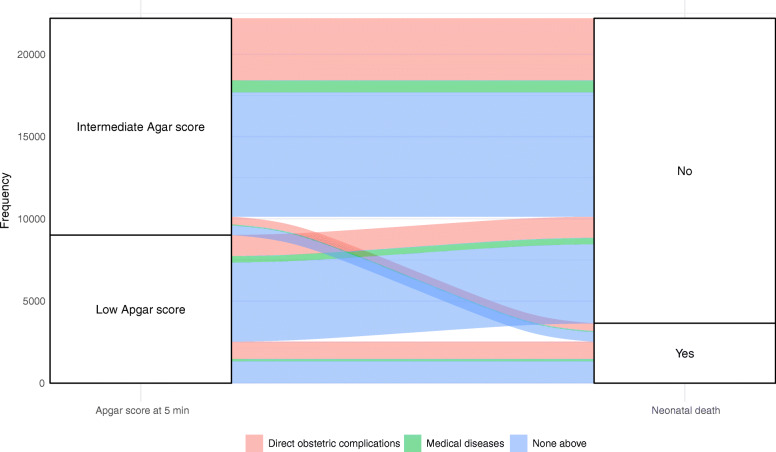
The associations of new-borns with low and intermediate Apgar scores at 5 min and neonatal deaths under different types of maternal complications

Among new-borns with low Apgar scores at 5 min whose mothers had direct obstetric complications, nearly half of them died (Fig. 1). Among new-borns with low Apgar scores whose mothers had medical diseases, the proportion of neonatal deaths was close to 1/3. Among neonatal deaths with low Apgar scores, almost half of their mothers had neither direct obstetric complications nor medical diseases. In the intermediate Apgar group, these proportions were much lower.

**Fig. 2:**
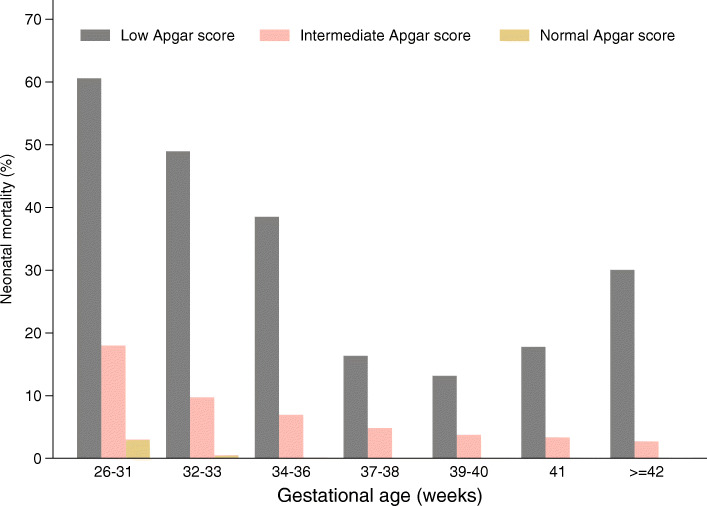
Weighted neonatal mortality in the Apgar score group at 5 min stratified by gestational age

The weighted neonatal mortality rate with a low Apgar score at 5 min was 28.72%, which was higher than that for births with an intermediate (8.28%) or with a normal Apgar score (0.06%). We examined the weighted neonatal mortality rates stratified by gestational age and Apgar score at 5 min (Fig. 2). The results showed that the neonatal mortality rate of births with a low Apgar score (0–3) was higher than that of births with a normal (7–10) or intermediate Apgar score (4–6) in each gestational age group. Among births with low Apgar scores (0–3), the neonatal mortality rate decreased progressively with gestational age (26–40 weeks) but increased if pregnancy was prolonged to over 41 weeks of gestation. This pattern was not the same as the trend among groups of new-borns with normal or intermediate Apgar scores, whose mortality rates continued to trend downward by gestational age. The preterm birth group with a low Apgar score at 5 min had the highest rate of death (52.29%) compared with the rates of the term birth group (14.67%) and post-term group (30.08%).

**Fig. 3:**
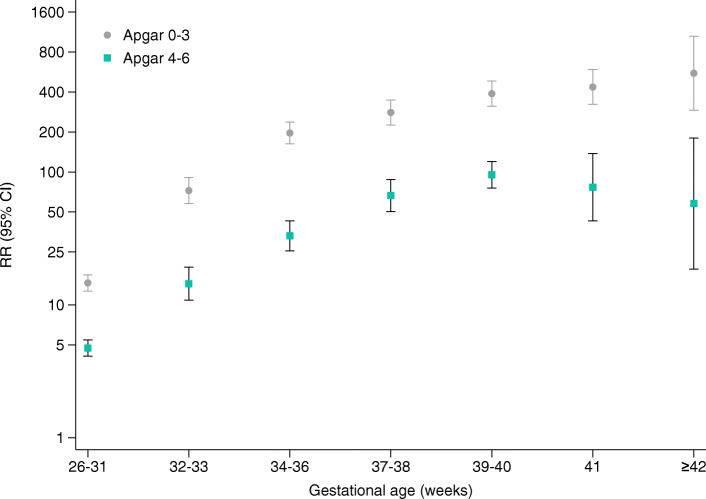
Adjusted RRs of Apgar score at 5 min for neonatal mortality stratified by gestational age. Notes: Adjusted RRs and 95% CIs of neonatal mortality for births with low (0–3) and intermediate (4–6) Apgar scores at 5 min compared with normal (7–10) Apgar scores at 5 min. RRs were adjusted for hospital level, maternal age, maternal education, maternal marital status, history of caesarean section, number of antenatal visits, neonatal sex, use of anaesthesia, mode of delivery, foetal presentation, birthweight categories, days that the mother stayed at the hospital, maternal complications and year of delivery (2012, 2013, 2014, 2015, 2016)

Apgar score stratified by gestational age at birth was strongly associated with neonatal mortality. Compared with those of a normal Apgar score at 5 min, the RRs of neonatal death associated with a low Apgar score at 5 min increased greatly and progressively with advancing gestational age after adjustment for several related factors. The peak was apparent at gestational ages of 42 weeks or above (Fig. 3). In contrast, the RRs associated with intermediate Apgar scores at 5 min after birth peaked at 39–40 weeks of gestation and subsequently decreased as the gestational age increased to 42 weeks or above while remaining statistically significant.

Because the proportion of the birthweight group varied with each Apgar-score group at 5 min (Table 1), we stratified the analysis by SGA categories to further examine the association between neonatal mortality and Apgar score at 5 min among the different gestational age groups. As shown in Table 2, in both the preterm and term new-born groups, babies who were not SGA had a lower mortality rate than those that were SGA, within each Apgar score group at 5 min. Regarding of whether the birth was preterm with or without SGA or term with or without SGA, the neonatal mortality rate decreased with increasing Apgar score. Compared with that for neonatal mortality among births with normal Apgar scores, the adjusted RR for neonatal mortality among births with low Apgar scores was 43.96 (95% CI 36.98–52.26) in the group of preterm births that were SGA, which was much lower than that in the group of term births that were not SGA (adjusted RR 392.76, 95% CI 318.69–484.03). The same results were found in the intermediate Apgar score group. When the global INTERGROWTH-21st standards were used to define the SGA group, similar results were found. The biggest difference between the results of these two standards was that the number of SGA cases calculated based on the global INTERGROWTH-21st standards was less than that calculated by Chinese standards.

**Table 2:** Neonatal mortality stratified by gestational age groups and birthweight categories among different Apgar score groups at 5 min

Standards	Gestation age (weeks)	Weight birth by gestational age	Low Apgar score		Intermediate Agar score		Normal Apgar score	
			No. of death (rate per 100 births^**a**^)	Adjusted relative risk^**b**^ (95%CI)	No. of death (rate per 100 births^a^)	Adjusted relative risk ^**b**^ (95%CI)	No. of death (rate per 100 births^**a**^)	Adjusted relative risk ^**b**^ (95%CI)
Chinese standards	< 37	with SGA	480 (55.76)	43.96 (36.98–52.26)	263 (15.87)	13.47 (11.15–16.26)	475 (0.99)	1 (reference)
		without SGA	1212 (49.96)	91.67 (79.84–105.24)	558 (11.74)	22.90 (19.79–26.49)	1267 (0.40)	1 (reference)
		Total$	1692 (51.48)	75.83 (66.85–86.01)	821 (12.79)	20.33 (17.89–23.11)	1742 (0.47)	1 (reference)
	≥37	with SGA	200 (30.41)	299.14 (240.94–371.40)	83 (6.45)	61.03 (45.02–82.73)	340 (0.08)	1 (reference)
		without SGA	501 (12.22)	392.76 (318.69–484.03)	164 (3.34)	97.47 (78.47–121.09)	1711 (0.03)	1 (reference)
		Total^c^	701 (14.64)	373.15 (310.76–448.07)	247 (3.92)	87.23 (72.44–105.05)	2051 (0.03)	1 (reference)
The global Intergrowth-21st standards	< 37	with SGA	338 (60.82)	35.22 (28.94–42.85)	189 (18.92)	11.45 (9.23–14.19)	290 (1.38)	1 (reference)
		without SGA	1354 (49.58)	86.41 (75.90–98.36)	632 (11.67)	21.86 (19.06–25.06)	1452 (0.41)	1 (reference)
		Total^c^	1692 (51.48)	75.09 (66.12–85.29)	821 (12.79)	20.18 (17.75–22.94)	1742 (0.47)	1 (reference)
	≥37	with SGA	187 (34.22)	310.31 (248.46–387.56)	82 (7.19)	64.35 (47.44–87.28)	274 (0.09)	1 (reference)
		without SGA	508 (12.14)	380.54 (308.81–468.94)	162 (3.21)	91.27 (73.53–113.29)	1772 (0.03)	1 (reference)
		Total^c^	695 (14.60)	368.90 (307.54–442.50)	244 (3.91)	84.15 (69.97–101.20)	2046 (0.03)	1 (reference)

^**a**^ Weighted for sampling distribution of the population

^**b**^ RRs were adjusted for hospital level, maternal age, maternal education, maternal marital status, history of caesarean section, number of antenatal visits, neonatal sex, use of anaesthesia, mode of delivery, foetal presentation, days that the mother stayed in the hospital after delivery, maternal complications and year of delivery (2012, 2013, 2014, 2015, 2016)

^**c**^ RRs were adjusted for hospital level, maternal age, maternal education, maternal marital status, history of caesarean section, number of antenatal visits, neonatal sex, use of anaesthesia, mode of delivery, foetal presentation, birthweight by gestational age, days that the mothers stayed at the hospital after delivery, maternal complications and year of delivery (2012, 2013, 2014, 2015, 2016)

A low Apgar score at 5 min was strongly associated with neonatal mortality (adjusted RR 126.50, 95% CI 107.35–149.06), and an intermediate Apgar score at 5 min was also associated with neonatal mortality (adjusted RR 30.27, 95% CI 26.11–35.10). However, the effect of the Apgar score was even stronger when scores at 1 and 5 min were combined (Table 3). Compared with that in the groups with scores of 7–10 at both 1 and 5 min, the risk for neonatal death increased by over 200-fold in both groups with scores of 3 or lower. Even new-borns who recovered from a low Apgar score at 1 min to a normal Apgar score at 5 min had a nearly 13-fold increased risk of neonatal death compared with those with normal scores at both 1 and 5 min.

**Table 3:** Relative risks of Apgar score at 1 and 5 min for neonatal mortality

	1-min Apgar score	5-min Apgar score	Total No. of livebirths	No. of deaths (rate per 100 births^**a**^)	Adjusted relative risk^**b**^ (95%CI)
Total	0–3	0–3	5039	2292 (46.58)	220.13 (184.91–262.07)
	0–3	4–6	5822	511 (8.40)	47.42 (40.11–56.08)
	0–3	7–10	6481	122 (1.61)	12.49 (9.69–16.11)
	4–6	0–3	454	187 (42.51)	160.43 (128.41–200.44)
	4–6	4–6	5959	511 (8.37)	42.75 (35.93–50.85)
	4–6	7–10	40,239	447 (1.01)	9.18 (7.82–10.79)
	7–10	0–3	3514	46 (1.33)	28.25 (19.25–41.46)
	7–10	4–6	1399	94 (7.35)	48.08 (36.64–63.09)
	7–10	7–10	6,485,078	3318 (0.05)	1(reference)
Hospital level 1	0–3	0–3	239	100 (41.38)	400.16 (232.21–689.56)
	0–3	4–6	321	11 (3.34)	44.81 (18.93–106.11)
	0–3	7–10	356	1 (0.12)	1.93 (0.25–14.82)
	4–6	0–3	16	10 (64.94)	406.37 (183.13–901.73)
	4–6	4–6	323	16 (5.12)	56.39 (24.37–130.44)
	4–6	7–10	2981	12 (0.42)	8.40 (3.68–19.18)
	7–10	0–3	176	3 (1.91)	54.34 (14.23–207.47)
	7–10	4–6	53	1 (2.12)	27.99 (9.95–78.77)
	7–10	7–10	447,576	140 (0.03)	1(reference)
Hospital level 2	0–3	0–3	2090	1011 (48.59)	297.96 (238.97–371.50)
	0–3	4–6	2408	190 (7.83)	60.27 (47.76–76.05)
	0–3	7–10	2584	32 (1.12)	11.35 (7.12–18.10)
	4–6	0–3	195	86 (44.65)	214.20 (161.22–284.60)
	4–6	4–6	2507	214 (8.31)	56.95 (45.55–71.21)
	4–6	7–10	18,769	157 (0.85)	10.53 (8.14–13.60)
	7–10	0–3	1740	20 (1.16)	25.61 (14.02–46.79)
	7–10	4–6	545	48 (9.18)	71.98 (51.20–101.19)
	7–10	7–10	3,121,012	1427 (0.05)	1(reference)
Hospital level 3	0–3	0–3	2546	1101 (44.41)	126.40 (101.41–157.56)
	0–3	4–6	2888	295 (10.46)	32.91 (26.11–41.49)
	0–3	7–10	3175	84 (2.62)	12.00 (8.53–16.89)
	4–6	0–3	222	79 (35.26)	80.30 (57.87–111.42)
	4–6	4–6	2933	258 (8.90)	26.89 (21.44–33.72)
	4–6	7–10	16,820	260 (1.51)	7.23 (6.03–8.68)
	7–10	0–3	1422	21 (1.58)	27.92 (17.40–44.79)
	7–10	4–6	734	37 (4.91)	22.46 (15.01–33.62)
	7–10	7–10	2,586,911	1580 (0.06)	1(reference)

^**a**^ Weighted for sampling distribution of the population

^**b**^ RRs were adjusted for hospital level, maternal age, maternal education, maternal marital status, history of caesarean section, number of antenatal visits, neonatal sex, use of anaesthesia, mode of delivery, foetal presentation, birthweight categories, gestational age categories, days that the mother stayed at the hospital after delivery, maternal complications and year of delivery (2012, 2013, 2014, 2015, 2016)

## Discussion

In our analysis, 7633 deaths at 26 or more completed weeks of gestation were reported in 438 health facilities between 2012 and 2016, giving a weighted neonatal mortality of 1.1 per 1000 live births, which was similar to the rate reported in the UK [9]. Among births between 26 and 36 weeks of gestation, the adjusted overall neonatal mortality was 1.18%, similar to the rate estimated in Dallas, TX, USA (1.02%) [20].

As a value that quantifies the effects of obstetric anaesthesia, the 10-point Apgar score, regardless of underlying cause, has been routinely used worldwide for more than 60 years, since 1953, to quickly and summarily assess the condition and prognosis of every new-born child [6]. The Apgar score at 5 min after birth has been used more widely as an index of the early neonatal condition than the 1-min Apgar score [21]. Despite the warning against overinterpretation of the score for predicting children’s outcomes that has been in place since the Apgar score was proposed [6], associations between the Apgar score and short-term or long-term health outcomes have still been reported [9, 20]. Several studies present an opinion that the Apgar score is antiquated because of the dramatic changes in the care of new-borns over the past 60 years, but studies have still found that the Apgar score is useful for evaluating the risk of neonatal death clinically [22, 23]. With advances in technology, there are indeed some more accurate assessment methods, such as blood pH, umbilical cord arterial lactate, base excess (BE) and other indicators that reflect metabolic acidosis. However, these advanced indicators are not available in all hospitals in China. Only a small number of high-level hospitals (Level 3 hospitals) can provide these advanced tests. However, a large number of low-level hospitals (Level 1 and Level 2 hospitals) are unable to use these advanced indicators. In addition, it takes a long time to obtain the results of these indicators. The advantage of the Apgar score over these advanced indicators is that it is immediately available on site, and the results based on the score can also be used for timely intervention treatment. Therefore, the Apgar score has been used clinically to guide neonatal resuscitation. Given that the Apgar score does have some subjectivity, it should be assessed by both the obstetrician and the neonatologist to improve the accuracy.

In China, the Apgar score has been used for many years and has become a routine assessment that obstetrics specialists need to perform immediately after childbirth. It is important to note that although the overall mortality rates among neonates born before term are close to those of high-income countries, the neonatal mortality rates, in both the low- and intermediate-Apgar score groups, are much higher in our study (524 per 1000 live births and 132 per 1000 live births, respectively) than those of high-income countries [20]. On the other hand, there is still a gap between China and high-income countries in terms of the sustained treatment of preterm infants with low Apgar scores. Another possible and unavoidable reason is the poor long-term outcomes of preterm infants with low Apgar scores and limited family economics; family members are more likely to give up treatment for these new-borns in China. With the growing number of births in China since the introduction of the universal two-child policy in October 2015 [24], the Apgar score may still be a useful indicator for rapidly predicting the risk of death in the neonatal period.

It is generally accepted that neonatal mortality is associated with gestational age [25], and preterm birth accounts for 75% of perinatal deaths [26]. In our analysis, the proportion of neonates with low Apgar scores at 5 min decreased rapidly from 17.57% at 26 completed weeks of gestation to 0.12% at 37 weeks (Additional file 1). This result is consistent with a previous finding indicating that births before 37 weeks of gestational age usually have a higher frequency of low Apgar scores at 5 min [27]. It is necessary to note that neonatal mortality related to Apgar score is influenced by gestational age and that the effect of gestational age is different between the low and intermediate Apgar score groups. There is no doubt that the decreased Apgar score is related to the increased risk of neonatal mortality in both gestational age groups. However, the relative risk of an intermediate Apgar score for neonatal mortality decreased after 40 completed weeks of gestational age; conversely, the relative risk of a low Apgar score for neonatal mortality subsequently increased. The association between neonatal mortality and Apgar score observed in our study is not consistent with the findings of the UK study [9], meaning that both of relative risk values of the low and intermediate Apgar score groups decreased after 41 weeks of gestational age. This may suggest that the treatment of new-borns with low Apgar scores at 5 min and over 40 completed weeks of gestational age in China is less effective than that in high-income countries. In addition, pregnancy termination is more common at a gestational age over 41 weeks in China according to clinical guidelines [28]. This means that in medical institutions in China, most post-term births are due to a lack of regular antenatal care, which may lead to a higher proportion of new-borns with underlying diseases among post-term births. Neonates with underlying diseases such as congenital abnormalities, meconium staining of the amniotic fluid or acidosis at birth often have low Apgar scores as well as higher mortality rates [29].

The distribution of Apgar scores is related to gestational age, and babies born before 37 weeks of gestation are at an increased risk of neonatal mortality. However, preterm births include groups with or without SGA, and SGA is an important cause of foetal and neonatal mortality [30, 31]. Previous studies stratified the risk of neonatal death or short- and long-term adverse health problems in relation to low Apgar score at 5 min by either birthweight or weeks of gestation [9, 22, 27, 32], or only evaluated the neonatal mortality in the combined presence of preterm birth (PTB) and SGA [25, 33, 34], despite the strong association between weeks of gestation and weight at birth that had been reported widely and were both principal health indicators of newborns. However, few studies have stratified the risk of neonatal mortality in relation to the Apgar score at 5 min by the combination of gestational age and birthweight by gestational age. Our study showed that when birth occurred at a higher gestational age (term) and under better birthweight conditions (without SGA) and if the neonate also had a low Apgar scores, the risk of neonatal mortality increased compared with that of a neonate with a normal Apgar score. Sensitivity analysis using the global INTERGROWTH-21st standards further confirmed this association. This result suggested that for births with a good gestational age and birthweight, there might still be some other risk factors, such as birth defects. The Apgar score was still a meaningful predictor for the adverse outcomes of these new-borns. We should never neglect the care of any new-born with a poor Apgar score, even when the neonate’s gestational age and birthweight are not poor.

Changes in Apgar score values at different times were used to assess the short-term and long-term risks of adverse outcomes [8, 35, 36]. A population-based study of term infants in Norway reported that the effect of Apgar score was even stronger when scores at 1 and 5 min were combined: if both scores were 3 or lower, the risks for neonatal death and infant death increased 642-fold and 123-fold, respectively, compared with scores of 7 to 10 [8]. However, in this study, no neonatal death was recorded when the Apgar score was 4–6 or 7–10 at 1 min but fell to 0–3 at 5 min, and the relative risks among all groups could not be compared. Our analysis confirmed that the neonatal mortality risk was higher for new-borns with low Apgar scores at both 1 min and 5 min (though no more than 0.1% births drop into the group) than for those with low Apgar scores only at 5 min. We were also surprised to find that the neonatal death risk was higher among new-borns whose Apgar scores fell from 7 to 10 at 1 min to 4–6 at 5 min than among new-borns whose Apgar scores fell from 7 to 10 at 1 min to 0–3 at 5 min. We further stratified the analysis by hospital level and found that the same results were found only in Level 2 hospitals. This result suggested that Level 2 hospitals in China may have not paid enough attention to the treatment and care of new-borns whose Apgar scores changed slightly but not very seriously.

The limitations of our study are as follows. (1) In the NMNMSS, infants were followed up from birth until their mothers were discharged from the hospitals, and the longest time for monitoring was less than 42 days, the standard time for postpartum women. All infant deaths recorded in the surveillance system occurred before maternal discharge; however, data on the exact time of death were not collected in the NMNMSS. Therefore, the neonatal mortality rate in our study is actually the neonatal mortality rate before maternal discharge from hospitals. (2) As the NMNMSS did not collect information on neonatal diseases (such as birth defects), adjustments for these factors cannot be made in the models used, so the relationship between Apgar score and infant mortality should be interpreted with caution.

## Conclusions

This study is the first to analyse the relationship between Apgar scores and neonatal mortality in more than 400 hospitals and over 6 million live births in China. This suggests that the Apgar score is not only meaningful for preterm infants but also useful for term infants, especially for term infants who are not SGA, even if only a few term births had Apgar scores of 3 or less. When the new-born’s Apgar score worsened at 5 min compared with that at 1 min, regardless of whether the range was large or small, timely intervention was needed. There is still a gap between China and high-income countries in the sustained treatment of new-borns with low Apgar scores.

## Supplementary Information

**Additional file 1.** Distribution of Apgar score groups at 5 min by gestational age.

## Data Availability

The datasets generated and/or analysed during the current study are not publicly available due to the terms of our contract with the Chinese National Health Commission but are available from the corresponding author on reasonable request.

## Abbreviations

U5MR: Under-Five Mortality Rate
SGA: Small for gestational age
NMNMSS: National Maternal Near Miss Surveillance System
HELLP: Haemolysis, elevated liver enzymes and low platelets
HIV: Human immunodeficiency virus
AIDS: Acquired immunodeficiency syndrome
CI: Confidence interval
UK: United Kingdom of Great Britain and Northern Ireland
USA: United States of America
PTB: P-reterm birth
