# Supplementary figures and images for: Apgar score and neonatal mortality in China: an observational study from a national surveillance system
Source: BMC Pregnancy Childbirth. 2021 Jan 12;21:47. doi: 10.1186/s12884-020-03533-3 (PMC7802282; doi:10.1186/s12884-020-03533-3)

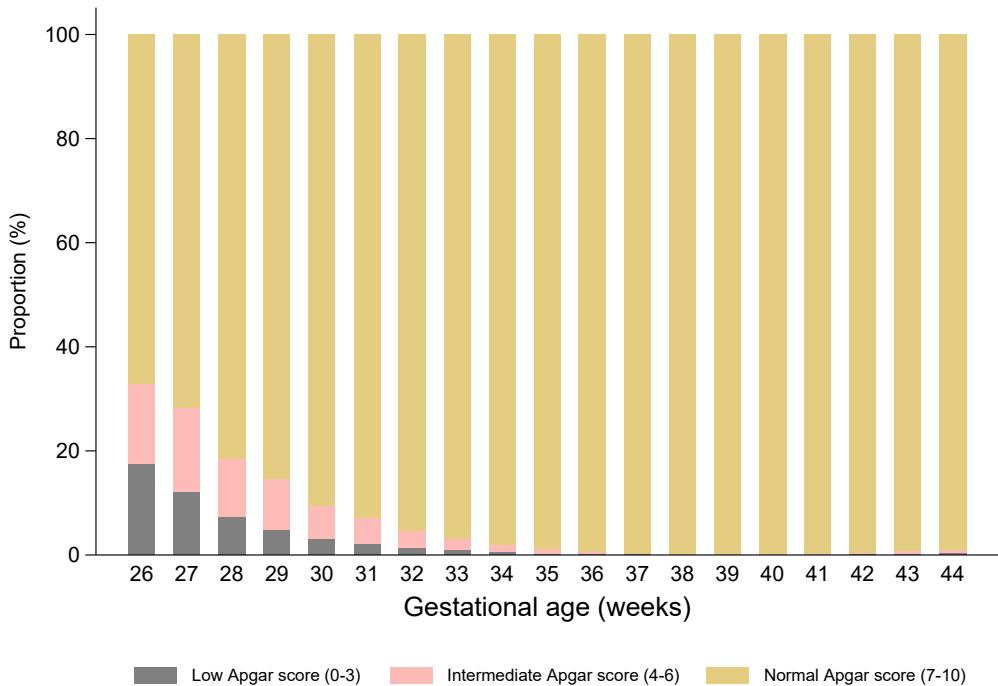

Supplement: Supplementary file 1 — Additional file 1. Distribution of Apgar score groups at 5 min by gestational age. [file 12884_2020_3533_MOESM1_ESM.pdf]
